# Effects of Clinical Pathways on Cesarean Sections in China: Length of Stay and Direct Hospitalization Cost Based on Meta-Analysis of Randomized Controlled Trials and Controlled Clinical Trials

**DOI:** 10.3390/ijerph18115918

**Published:** 2021-05-31

**Authors:** Dan Lin, Chunyang Zhang, Huijing Shi

**Affiliations:** 1 Department of Maternal, Child and Adolescent Health, School of Public Health, Fudan University, Shanghai 200032, China; dlin19@fudan.edu.cn; 2 Fujian Center for Disease Control and Prevention, Fuzhou 350001, China; jgbuyer@126.com

**Keywords:** clinical pathway, cesarean section, length of stay, direct hospitalization cost, health economics, meta-analysis

## Abstract

The cesarean section (CS) on maternal request increased sharply in China, bringing pressure to medical resources and national insurance. We assessed the use of clinical pathways (CPWs) for CS compared with conventional medical care by outcomes of length of stay (LOS) in hospital and direct hospitalization cost (DHC). Four Chinese electronic databases, including China National Knowledge Infrastructure (CNKI), Wanfang, CQVIP, and SinoMed, were explored to December 2020 for the full-text papers published in Chinese. Literature that quantitatively assessed the effects of CPW on LOS or DHC were eligible for inclusion. The weighted mean differences (WMDs) were pooled. Twenty-five articles were included in our analysis, with a total sample of 7761 women. These studies were performed from 2004 to 2017 and reported from 2005 to 2018. The synthesized results showed a shorter LOS (in days) (WMD = −1.37, 95% CI: −1.48 to −1.26) and a less DHC (CNY¥) (WMD = −520.46, 95% CI: −554.06 to −503.63) in the CPW group, comparing with that of conventional care. With the need for CS on the rise, the introduction of CPW could effectively reduce LOS and DHC, thereby releasing the medical resources and insurance pressure.

## 1. Introduction

Cesarean section (CS) could save mother’s and infant’s lives and should be universally accessible [1]. Over the past few decades, the CS rate in China increased steeply in all socio-economic groups and all levels of hospitals. A study suggested that the rate of CS from “maternal request” has notably increased to two fifths approximately [2]. Fear or anxiety of child-delivery or mental pressure during birth seems to be the most significant cause of CS [3]. Wealthy and better-educated women prefer cesarean delivery over nature birth since CS is free of pain and anxiety [4,5]. Although CS is a beneficial treatment from the patients’ perspective, the total expenditure has increased sharply and burdens the medical resource and insurance [5,6,7,8,9]. Therefore, there is a challenge to control resources and costs without affecting the quality of patient care [10,11].

Clinical pathways (CPWs), also known as care pathway or critical pathway, is a prominent organizational strategy to cut down expenditure and improve patient management [11,12,13,14,15,16,17]. It is described as complicated involvements that include the best available evidence and guidelines for a particular situation, including many elements [18,19]. Meanwhile, it is a multidisciplinary care plan that outlines the sequence and timing of actions required to achieve the desired patient outcomes and organizational goals related to quality, cost, patient satisfaction, and efficiency [20,21]. It aims to minimize latency and maximize resource utilization and care quality [18,19]. Compared with conventional medical care, CPW might be an appreciated approach to reduce the burden of both healthcare resources and insurance expenditure.

Primary studies invariably reported data with a focus on the length of stay (LOS) or direct hospitalization cost (DHC) rather than a comprehensive economic assessment [19,22]. Hence, the economic effect on CPW of CS is under debate in China since there were inconsistent results among individual studies. Therefore, this meta-analysis systematically evaluates the evidence for the economic effect on CPW of CS with LOS and DHC outcome measures compared to conventional medical care.

## 2. Materials and Methods

### 2.1. Eligibility Criteria

Paper that met those listed eligible criteria was selected: (1) All participants were pregnant women bearing a singleton, full-term, live fetus and all women have no or minor maternal and fetal complications during pregnancy and childbirth, which did not affect the implementation of CS. (2) All CSs were on maternal requests, which was interpreted as an intended optional CS without medical indications, and mothers with emergency CSs were not included [3]. (3) All included papers compared the CS provided by CPW to conventional diagnosis and treatment. (4) There were no less than one of the indicants in the study: (a) LOS, which was characterized as the number of days of hospitalization from admission to discharge; (b) DHC, which referred to accurate total hospital costs such as operation, intensive care units, medicines and consumable materials [11]. (5) Randomized controlled trials (RCTs) and controlled clinical trials (CCTs) (studies were considered to be randomized when the authors specifically stated that in the text, although random methods were not always fully described).

Papers were excluded if they were (1) illustrative (review papers, systematic evaluations, experimental studies, or case reports), (2) articles without the group of control, (3) articles did not evaluate at least one of the outcomes, (4) articles of non-CS surgeries, or (5) no estimates of both means and standard deviations (*SD*s) were reported simultaneously. If duplicated data were found, the study with the lengthiest observation period would be included.

### 2.2. Information Sources and Search Strategy

Studies about the effect of CPWs on LOS and DHC of CS in China were searched for in the databases of China National Knowledge Infrastructure (CNKI), Wanfang, CQVIP, and SinoMed for articles published in Chinese. Following the Preferred Reporting Items for Systematic Reviews and Meta-Analyses (PRISMA) guidelines [23], a systematic literature search in these electronic databases was developed. Without time limits, the search was up to 31 December 2020, to identify articles that reported means and *SD*s about LOS and DHC regarding CPW and conventional care of CS.

### 2.3. Search and Study Selection

**Figure 1:**
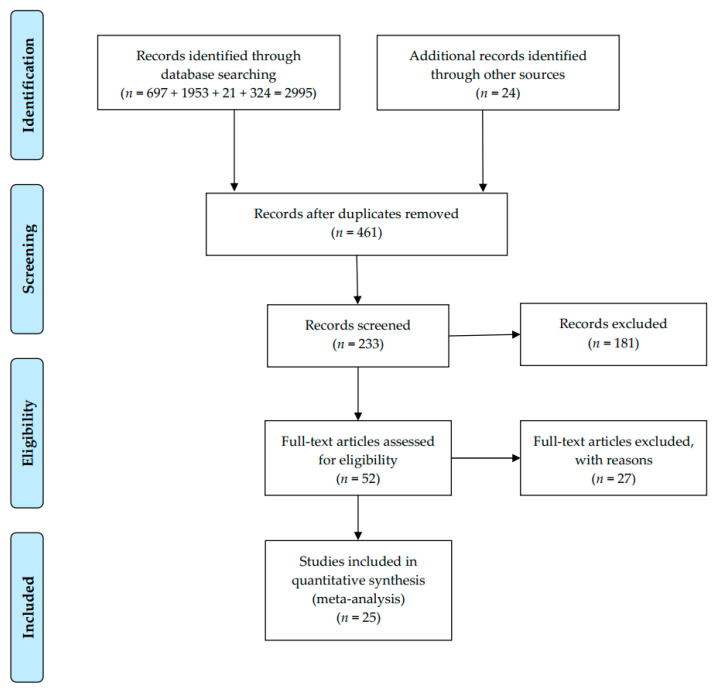
PRISMA flow diagram for meta-analysis.

The search approach was initially designed for the SinoMed and then applied to the others. Medical subject headings (MeSHs) terms were used to search for articles related to CPWs and CS: (1) cesarean section, (2) critical pathways, (3) length of stay, and (4) hospital costs or hospital charges. Meanwhile, a non-MeSH search was carried out in terms of the search string: (‘clinical pathway’ OR ‘critical pathway’ OR ‘clinical path’ OR ‘multidisciplinary approach’) AND (‘hospital day*’ OR ‘hospital time*’ OR ‘length of hospital stay’ OR ‘length of stay’) AND (cesarean); (‘clinical pathway’ OR ‘critical pathway’ OR ‘care map’ OR ‘clinical path’ OR ‘multidisciplinary approach’) AND (‘hospital cost*’ OR ‘hospital charge*’) AND (cesarean). Manually searching for references for eligible studies was conducted to find other related papers. The selection procedure was displayed in Figure 1.

### 2.4. Statistics Items and Data Collection Process

Through standardized data collection forms, two reviewers carried out the data extraction independently. A third reviewer confirmed all data, and all discrepancies were resolved by group discussion. Extracted items include title, first author, year of publication, geographic area, research design, sample size, and the targeted estimates (i.e., mean and *SD*). Data duplication in different papers was eliminated, and the study with the most informative data would be included.

### 2.5. Risk of Bias Assessment

Evidence quality was assessed by the Cochrane Collaboration’s Tool for risk of bias [24]. This tool judges the risk of bias as “low”, “unclear”, and “high” risks for six domains. An article with low risk was defined as high-quality research, and that with high risk was classified as low-quality research. The quality of each paper was evaluated by two reviewers separately. Any disagreements were resolved through consensus.

### 2.6. Summary Measures

The outcomes to measure the economic effect for CPWs of CS in the included studies were the weighted mean differences (WMDs) of the LOS and DHC. WMD was a comprehensive indicator of LOS or DHC difference between CPW and conventional care groups [11]. When comparing the DHC of CPW to conventional care, costs are calculated in the Chinese Yuan (CNY¥). Costs were actualized to 2018 values using a rate of inflation based on the evolution of the Consumer Price Index, which was released from the State Statistical Bureau of China.

### 2.7. Synthesis of Results and Data Analysis

Data analysis abided by the Cochrane Collaboration Guideline [25]. For continuous variables in this meta-analysis, the WMD and 95% confidential interval (CI) were used. The weighting process considers the variance near the mean to calculate the study’s contribution to the overall result. As the mean is affected by extreme values, the analysis can only use the mean if a standard deviation is provided. The *p*-value less than 0.05 was utilized as the significance threshold.

Heterogeneity would be recognized and confirmed by the Q test, *I*^2^ statistic, H statistic, and the Galbraith plot. A Q-statistic calculation of a *p*-value less than 0.05 would suggest heterogeneity in meta-analyses [26]. The *I*^2^ statistic was applied to analyze heterogeneity extent [27]. The more considerable value of *I*^2^ indicates the heterogeneity. The Cochrane handbook [28] defined that an *I*^2^ between 30% and 60% represented moderate heterogeneity and a value of *I*^2^ between 50% and 90% represented substantial heterogeneity. The H statistic quantified heterogeneity in this meta-analysis as it seems that values above 1.5 may cause considerable caution, while values below 1.2 may cause no concern [29]. Galbraith plots show the degree of heterogeneity through vertical scatter plots of points [30]. The “random effects” approach was applied, which is designed to process statistics from a single study [11]. Results were presented by forest plots, which displayed the individual estimates.

If the high heterogeneity was clearly defined, source exploration was conducted by meta-regression, subgroup analyses, and sensitivity analyses. By dividing the analyses into subgroups, the robustness of the pooled results would be examined in subgroups (according to study design), and a random effect model would explore the heterogeneity of within-study. Factors related to estimates (pooled WMDs) would be studied and the results reported as WMDs with 95% CIs. Meanwhile, a one-study removed approach would be employed to evaluate the stability and the implication of each study in the sensitivity analyses [31].

Publication bias was assessed by a set of funnel plots. The publication bias in each result of the meta-analysis was evaluated by visualization of asymmetry. However, as the subjective nature of graphical evaluation, the publication bias could not be confirmed. Hence, a contour-enhanced funnel plot was used to explore the source of bias further if an asymmetric funnel plot was identified. The addition of the contours of statistical significance was easier to evaluate the proportion of studies published in the meta-analysis at and around statistical significance [32,33]. We also studied the possibility of publication bias by carrying out meta-bias analyses (Begg’s tests and Egger’s tests). The trim-and-fill method was also applied to figure out the numbers of articles to be filled. All the analyses were conducted by the software of StataSE-64. All *p*-values were two-tailed, and a *p*-value < 0.05 indicated significance.

## 3. Results

### 3.1. Study Selection and Characteristics

The literature search obtained 233 related studies. Totally, 181 were dropped after the title or abstract reading, leaving 52 for full-text reading. Eventually, 25 studies (15 RCTs and 10 CCTs) met the criteria and evaluated the LOS or DHC between CPW and conventional care (Figure 1, Table 1) [34,35,36,37,38,39,40,41,42,43,44,45,46,47,48,49,50,51,52,53,54,55,56,57,58]. Twelve studies concerned the LOS, seven for DHC and six for both. These studies were conducted between 2004 and 2017 in different provinces across China and reported between 2005 and 2018. A total sample of 7761 patients was included, and data of them were reported. The characteristics of the literature were summarized in Table 1.

### 3.2. Risk of Bias within Studies (Quality Assessment)

The quality of evidence for included studies was shown in Figures S1 and S2 in the Supplementary Materials. In many studies, the randomization process was described, and the meaning of conventional care (control group) was demonstrated clearly to help in the assessment. However, all studies were concerned about bias, mainly because there are no reports on whether random allocations are hidden. Since the practice of CPW was not double-blinded to allocation, all the health care workers knew clearly which group of patients were in CPW.

### 3.3. Effect of CPW: LOS

**Figure 2:**
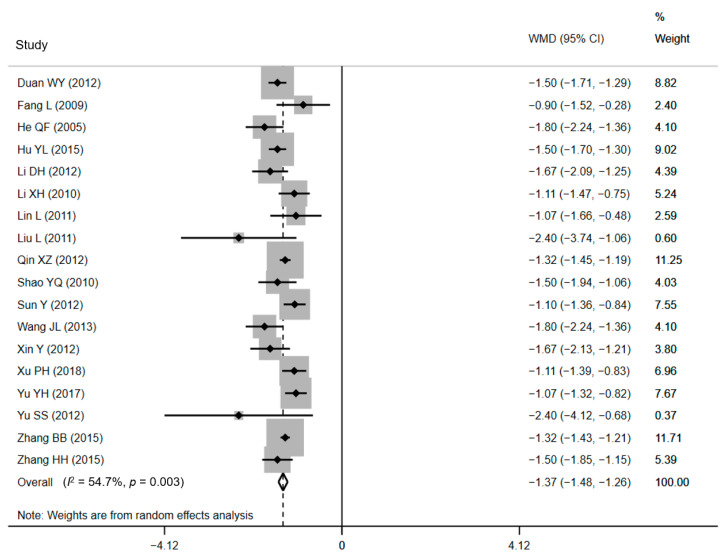
Forest plot of effects on LOS. Abbreviations: WMD, weighted mean difference; CI, confidential interval; LOS, length of stay.

Data of average LOS (ALOS) were identified from 18 studies [35,36,38,39,41,42,43,44,46,47,48,49,51,52,54,55,56,57]. Ten RCTs and eight CCTs consisting of a sample size of 6871 women examined the effect of CPWs on the ALOS, and 16 of them displayed critical effects. The ALOS ranged from 4.21 ± 0.83 days to 7.50 ± 1.80 days in CPW groups and from 5.53 ± 0.92 days to 8.92 ± 1.86 days in control groups. The ALOS data were pooled, and a notably shorter ALOS in CPW groups was noticed in the outcomes (WMD = −1.37 days, 95% CI: −1.48 to −1.26 days, *p* < 0.001). Since we expected low homogeneity, a DerSimonian and Laird random-effects model [59] was used to pool all estimates across researches. Expectedly, heterogeneity between studies reporting on ALOS was moderate based on the results of the Q test (Q = 37.56, *p* = 0.003) and *I*^2^ statistics (*I*^2^ = 54.7%, *p* < 0.001), respectively. The forest plot is shown in Figure 2. The H value was 1.5 (95% CI: 1.1–1.9, *p* = 0.003), which demonstrated the heterogeneity, too. Nevertheless, heterogeneity was not recognized by the Galbraith plot as the estimates of all studies positioned between the two parallel straight lines that defined the area of the 95% CI [30] and were not near the origin (Figure S3 in the Supplementary Materials).

### 3.4. Effect of CPW: DHC

**Figure 3:**
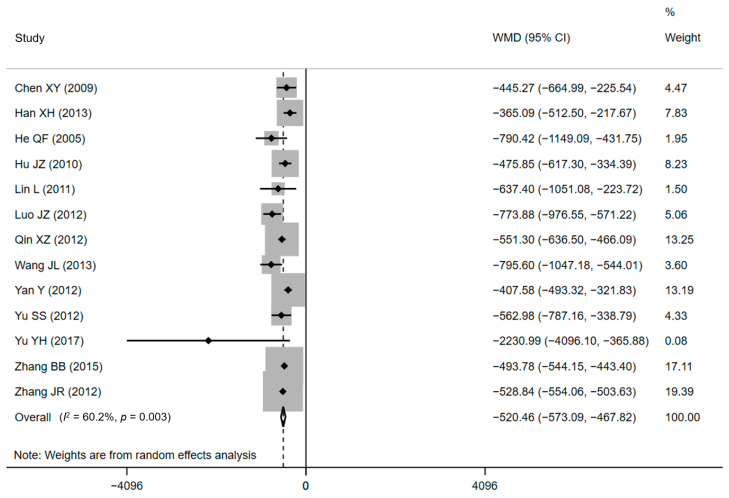
Forest plot of effects on DHC. Abbreviations: WMD, weighted mean difference; CI, confidential interval; DHC, direct hospitalization cost.

Thirteen of the included papers (eight RCTs and five CCTs), standing for a sample of 4642 treated women, declared cost effects [34,37,38,40,43,45,46,49,53,54,55,56,58]. All of the included studies found significantly lower DHC for CPW groups when comparing with conventional care. The average DHC ranged from CNY¥ 2774.46 ± 235.79 to CNY¥ 7745.01 ± 1172.43 in CPW groups and from CNY¥ 3182.04 ± 400.84 to CNY¥ 8408.16 ± 4198.46 in control groups. The pooled results showed that compared with conventional care, lower expenditure was associated with the use of the CPW, as shown in Figure 3 (WMD = CNY¥ −520.46, 95% CI: CNY¥ −554.06 to −503.63, *p* = 0.003). However, there was substantial heterogeneity existed in included studies based on the results of *Q* test (Q = 30.18, *p* = 0.003) and *I^2^* statistics (*I*^2^ = 60.2%, *p* < 0.001), respectively. The H value was 1.6 (95% CI: 1.2–2.1, *p* = 0.003) and demonstrated the heterogeneity, too. Nevertheless, heterogeneity was not recognized by the Galbraith plot as the estimates of all studies positioned between the two parallel straight lines that defined the area of the 95% CI [30] and were not near the origin (Figure S4 in the Supplementary Materials).

### 3.5. Sensitivity Analyses and Publication Bias

The pooled estimates for WMD of LOS ranged between−1.39 days (95% CI: −1.50 to −1.29 days, when the study by He QF [38] was excluded) and −1.35 days (95% CI: −1.45 to −1.25 days, when the study by Yu YH [54] was excluded). Meanwhile, the pooled estimates WMD of DHC ranged from CNY¥ −535.25 (95% CI: CNY¥ −482.46 to −588.04, when the study by Luo JZ [45] was excluded) to CNY¥ −505.54 (95% CI: CNY¥ −554.71 to −456.37, when the study by Yan Y [53] was excluded). The sensitivity analysis result indicated that no studies have inappropriately affected the pooled estimates (Figures S5 and S6 in Supplementary Materials), and the results of the meta-analysis were robust.

The standard funnel plot was plotted to explore whether the substantial statistical heterogeneity among studies in this meta-analysis was caused by publication bias (Figure S7 in the Supplementary Materials). For publication bias detection on LOS data, the visual inspection of the funnel plot has detected asymmetry and suggested the presence of publication bias. To explore for an interpretation, a contour-enhanced funnel plot was plotted (Figure S8 in the Supplementary Materials). There is a strong mention of asymmetry, signifying that the missing researches were on the right side of the plot. It manifested that most studies had high statistical significance (all *p* < 0.01). Taking this result, we can infer that publication bias was more likely to be the cause of asymmetric funnel plots. However, Egger’s test (*p* = 0.24) (Figure S9 in the Supplementary Materials) and Begg’s test (*p* = 0.43) did not discover any publication bias. The trim-and-fill method [60] figured out that no study needing to be filled and trimmed.

For publication bias detection for the data of DHC, visual inspection for funnel plots detected asymmetry and suggested the presence of slight publication bias (Figure S10 in the Supplementary Materials). The contour-enhanced funnel plot figured out a strong suggestion of asymmetry, signifying that there were researches missing on the right side of the plot (Figure S11 in the Supplementary Materials). That plot also demonstrated that all of these studies had statistical significance (all *p* < 0.01), suggesting that publication bias may be a reasonable explanation for the funnel’s asymmetry. However, Egger’s linear regression (*p* = 0.41) (Figure S12 in the Supplementary Materials) or Begg’s test (*p* = 0.20) did not identify statistical evidence for publication bias. Therefore, in this funnel plot, publication bias may not be the cause of the observed asymmetry, and publication bias in this study is unlikely to cause heterogeneity. Application of the trim-and-fill method [60] figured out that no study needing to be filled and trimmed. Furthermore, although the number of articles was small, the unpublished researches were unlikely to threaten the effectiveness of the original meta-analysis.

### 3.6. Meta-Regression and Subgroup Analyses

Heterogeneity among studies was further explored by meta-regression, and the covariate was the study design. Restricted maximum likelihood was performed to build the model of regression for WMD-covariate. Not a *p*-value less than 0.05 was detected for the study design of LOS, implying that the regression model for the covariate had no significance, and the design of the study might not be the source of heterogeneity in this meta-analysis for LOS. Noticeably, a *p* < 0.05 (*p* = 0.04) was found in the meta-regression of study design for DHC. The value of *I*^2^_res_ suggested that 55% of the residual variation is caused by heterogeneity, with the other 45% was attributable to within-study sampling variability. The value of adjusted *R*^2^ indicated that the difference of study design interprets 55% of the between-study variance.

**Figure 4:**
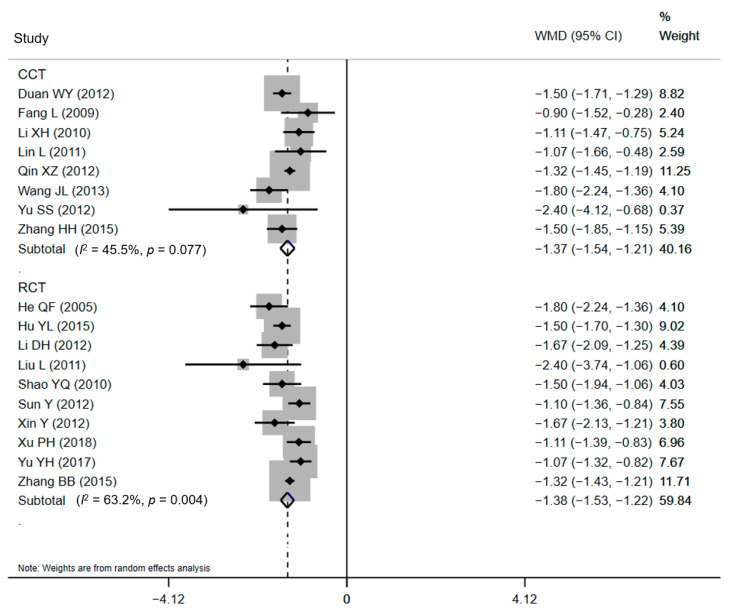
Forest plot of effects on LOS in different subgroups. Abbreviations: WMD, weighted mean difference; CI, confidential interval; LOS, length of stay.

**Figure 5:**
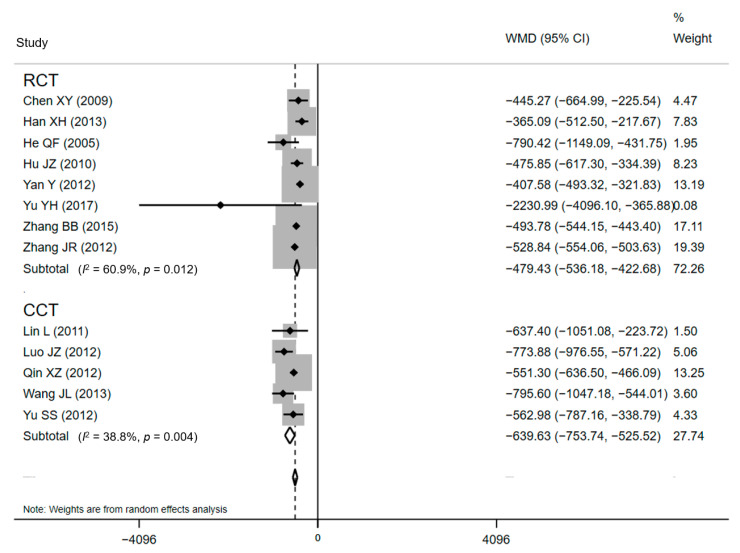
Forest plot of effects on DHC in different subgroups. Abbreviations: WMD, weighted mean difference; CI, confidential interval; DHC, direct hospitalization cost.

Subgroup analyses were developed to recalculate the pooled estimates in terms of study design (Table 2). The pooled effects for ten RCTs and eight CCTs were −1.38 days (95% CI: −1.53 to −1.22) and −1.37 days (95% CI: −1.54 to −1.21), respectively for LOS (Figure 4). The statistical pooling of the subgroups of RCTs was characterized by statistically inconsistent overall test values (*I*^2^ = 63.2%), which also reflects the heterogeneity. Similar results were detected in the polled effects for eight RCTs and five CCTs studies for DHC. The pooled WMDs were CNY¥ −479.43 (95% CI: CNY¥ −536.18 to −422.69) and CNY¥ −520.46 (95% CI: CNY¥ −573.09 to −467.83), respectively (Figure 5), and the studies in the subgroup of RCTs also demonstrated substantial heterogeneity (*I*^2^ = 60.9%). These substantial heterogeneities (63.2% and 60.9%, *p*-values < 0.05) pointed out that sampling errors did not cause the differences among studies in a subgroup. Between-subgroup heterogeneity was then investigated in the interaction tests, but no heterogeneity was found (Table 2).

## 4. Discussion

Nowadays, improvements in family income, access to health insurance, and higher women’s education level all accounted for the rise in CS on maternal request in urban and rural areas in China [5]. Furthermore, in pace with the relaxation of the one-child policy in China, the growth of the need for CS will emerge sharply and bring considerable challenge and burden for medical resources and national insurance [61,62,63]. Under the circumstances, this meta-analysis was conducted to collate economic data (LOS and DHC) for the CPW of CS. We proved that the introduction of CPW in China appeared, to some extent, to be valid in reducing LOS and DHC for CS, thereby releasing the burden of medical resources and the pressure of national insurance.

Decision-makers have enhanced the use of clinical practice guidelines and CPWs as a critical approach to advancing the effectiveness and efficiency of healthcare practices since 2009 [64,65]. More than 90% of public hospitals in China implemented CPWs, of which an average of 45 CPWs was implemented [65]. The National Health Commission of China promoted the implementation of CPWs [65], and many strategies have been utilized or recommended to promote physician compliance. The CPW showed a positive effect on LOS and DHC, and there are reasons to believe that the decrease in LOS and DHC in CS resulted from a superior organization and standardization of the process of care [65,66,67,68]. LOS and DHC are extensively used as measures of medical outcomes [20]. Still, we should bear in mind that they present health resources and funds and act as significant indexes for evaluating the quality of health care services. Although we did not analyze the patients’ satisfactions, it can be concluded that both the reduction in LOS and DHC can be attributed to a better quality of care [11,69,70,71]. These findings illuminate clinicians, hospital managers, policymakers, and researchers for their horizons upon the outcome of CPWs.

To the best of our knowledge, this meta-analysis is the first of its kind to comprehensively summarize the effects of practice for CPW of CS in the field of economic evaluation. The significant finding in the meta-analysis was associated with the positive impact of CPW. Our results are consistent with previous meta-analyses [11,19,20,69,72,73,74] for the significant reduction in LOS or DHC linked with the introduction of CPWs for many other clinical treatments/surgeries. All studies that measured ALOS or DHC reported results that supported CPWs. The pooled ALOS and DHC in hospitals displayed in these studies were significantly reduced when a CPW was introduced. A meta-analysis focused on CPW for endoscopic sinus surgery reported that compared to the conventional care and treatment, the CPW could effectively shorten the ALOS by a mean difference (MD) of two days and effectively reduce the per capita hospitalization costs [19]. Another meta-analysis for CPW in joint replacement practices specifically based on clinical trials reported that a shorter LOS in the CPW group was also noticed and less expense during a hospital stay related to the application of CPWs [11]. Additionally, in a recent meta-analysis that evaluated the implementation of CPWs in patients with gastrointestinal cancer, the synthesized results demonstrated a shorter ALOS by an MD of four days with CPW compared with conventional care, and the decrease in hospital costs was also associated with that [20]. According to these results, there is likely to be a tendency for the benefit of CPWs in inpatients treatment and have significant clinical and economic implications.

This meta-analysis may encourage more studies on this issue. Some uncertainties of CPW for CS require to be investigated in the future. Initially, the mechanism of how the CPW system work is still not explicit [19,20]. It is suggested to identify what crucial component(s) of CPW for CS could help the whole system work efficiently. Specifically, no single costs were analyzed in this meta-analysis to determine at which stage the costs could save. Most studies in the meta-analysis did not report whether reducing DHC accounts for the single charge of specific elements in the care process [11]. As a result, it is impossible to conclude that costs can be reduced through more appropriate care processes or simply by reducing overall hospital stays. Lastly, it would be interesting to investigate how hospital managers can integrate CPWs into the assessments of quality-improvement processes and how the development and implementation of CPWs inspire and encourage hospitals to improve practices for better outcomes [74,75].

Although CPW seems to reduce LOS and DHC in this meta-analysis effectively, caution is needed to interpret results because of limitations. The heterogeneity was substantial, and the results were without robustness. Hence, the generalizability of our findings requires caution. In addition, as with all meta-analyses or reviews, a limitation subjected to publication bias exists. Even for RCTs, authors prefer to report positive effects or trends in original articles when comparing a new technique to a standard one [11,19,76]. Meanwhile, studies of CPW for CS in China have demonstrated reductions in LOS and DHC. Still, small sample sizes used in some of the included studies might confine their capability to examine innovations for the result by methodological weaknesses [19]. In addition, from a methodological perspective, an interpretation bias might have occurred when the original articles of RCT mentioned randomness but did not offer a complete pathway to generate random sequences. Finally, since few studies reported concealed allocation schemes and blindness in surgical interventions, selection bias and implementation bias could not be ignored.

## 5. Conclusions

We summed up the evidence and evaluated the effect of CPW of CS on LOS and DHC in China. Results indicated that CPW could significantly reduce the LOS and DHC of CS. Therefore, CPW is strongly suggested to play a vital role in health economics. As a practical implementation, CPW should be strengthened in the clinical administration of CS in China. Further studies are encouraged to focus on the critical components of mechanisms within CPWs that can affect health care economics and even patient care outcomes.

## Figures and Tables

**Table 1:** Characteristics of studies included in this meta-analysis.

No.	Author	Publication Year	Study Period	Province	Study Design	No. of Participants	Risk of Bias
1	Chen XY [34]	2009	2008	Sichuan	RCT	248	Low
2	Duan WY [35]	2012	2011	Henan	CCT	320	High
3	Fang L [36]	2009	2007–2008	Hubei	CCT	120	High
4	Han XH [37]	2013	2012–2013	Xinjiang	RCT	755	Low
5	He QF [38]	2005	2004–2005	Guangdong	RCT	100	Low
6	Hu YL [39]	2015	2013–2014	Guangdong	RCT	280	Low
7	Hu JZ [40]	2010	2009	Guangdong	RCT	320	Low
8	Li DH [41]	2012	2010–2011	Hunan	RCT	164	Low
9	Li XH [42]	2010	2010	Jiangxi	CCT	300	High
10	Lin L [43]	2011	2010	Anhui	CCT	67	High
11	Liu L [44]	2011	2010–2011	Guangxi	RCT	133	Low
12	Luo JZ [45]	2012	2011–2012	Xinjiang	CCT	212	High
13	Qin XZ [46]	2012	2010–2011	Guangxi	CCT	560	High
14	Shao YQ [47]	2010	2009–2010	Shandong	RCT	280	Low
15	Sun Y [48]	2012	2010–2011	Guangdong	RCT	330	Low
16	Wang JL [49]	2013	2012–2013	Anhui	CCT	100	High
17	Wang Y [50]	2013	2012	Liaoning	CCT	108	High
18	Xin Y [51]	2012	2009–2011	Liaoning	RCT	205	Low
19	Xu PH [52]	2018	2016–2017	Anhui	RCT	320	Low
20	Yan Y [53]	2012	2011	Hunan	RCT	226	Low
21	Yu YH [54]	2017	2015–2017	Jiangsu	RCT	60	Low
22	Yu SS [55]	2012	2010	Jiangsu	CCT	626	High
23	Zhang BB [56]	2015	2013–2014	Liaoning	RCT	1000	Low
24	Zhang HH [57]	2015	2009–2012	Shanxi	CCT	567	High
25	Zhang JR [58]	2012	2011	Hebei	RCT	360	Low

Abbreviations: RCT, randomized controlled trials; CCT, controlled clinical trials.

**Table 2:** Results of subgroups analyses and tests of interaction.

Subgroup	Number of Studies	WMD	95% CI		*p*-Value	Tests for Heterogeneity		*p*-Value for Interaction
						*p*-Value (Q Statistic)	*I*^2^ (%)	
LOS								0.94
RCT	10	−1.38	−1.53	−1.22	<0.001	<0.001	63.2	
CCT	8	−1.37	−1.54	−1.21	0.08	<0.001	45.4	
DHC								0.30
RCT	8	−479.43	−536.18	−422.69	0.01	<0.001	60.9	
CCT	5	−520.46	−573.09	−467.83	0.16	<0.001	38.8	

Abbreviations: WMD, weighted mean difference; CI, confidential interval; LOS, length of stay; DHC, direct hospitalization cost; RCT, randomized controlled trials; CCT, controlled clinical trials.

## Data Availability

Not applicable.

## Supplementary Materials

The following are available online at https://www.mdpi.com/article/10.3390/ijerph18115918/s1, Figure S1: Summary chart of risk of bias assessment for included studies using the Cochrane risk of bias tool, Figure S2: Risk of bias presented as percentages across all included studies, Figure S3: Galbraith plot of effects on LOS, Figure S4: Galbraith plot of effects on DHC, Figure S5: Sensitivity analyses for assessing the impact of individual studies on the pooled estimate of LOS, Figure S6: Sensitivity analyses for assessing the impact of individual studies on the pooled estimate of DHC, Figure S7: Metafunnel funnel plot for the effect of LOS, Figure S8: Confunnel funnel plot of the effect on LOS, Figure S9: Egger’s linear regression of effect on LOS, Figure S10: Metafunnel funnel plot for effect of DHC, Figure S11: Confunnel funnel plot of effect on DHC, Figure S12: Egger’s linear regression of effect on DHC.
