# Supplementary material for: Effects of Clinical Pathways on Cesarean Sections in China: Length of Stay and Direct Hospitalization Cost Based on Meta-Analysis of Randomized Controlled Trials and Controlled Clinical Trials
Source: Int J Environ Res Public Health. 2021 May 31;18(11):5918. doi: 10.3390/ijerph18115918 (PMC8198843; doi:10.3390/ijerph18115918)
Supplement: Supplementary file 1 [file ijerph-18-05918-s001.zip › ijerph-1178169-supplementary.pdf]

Supplementary materials

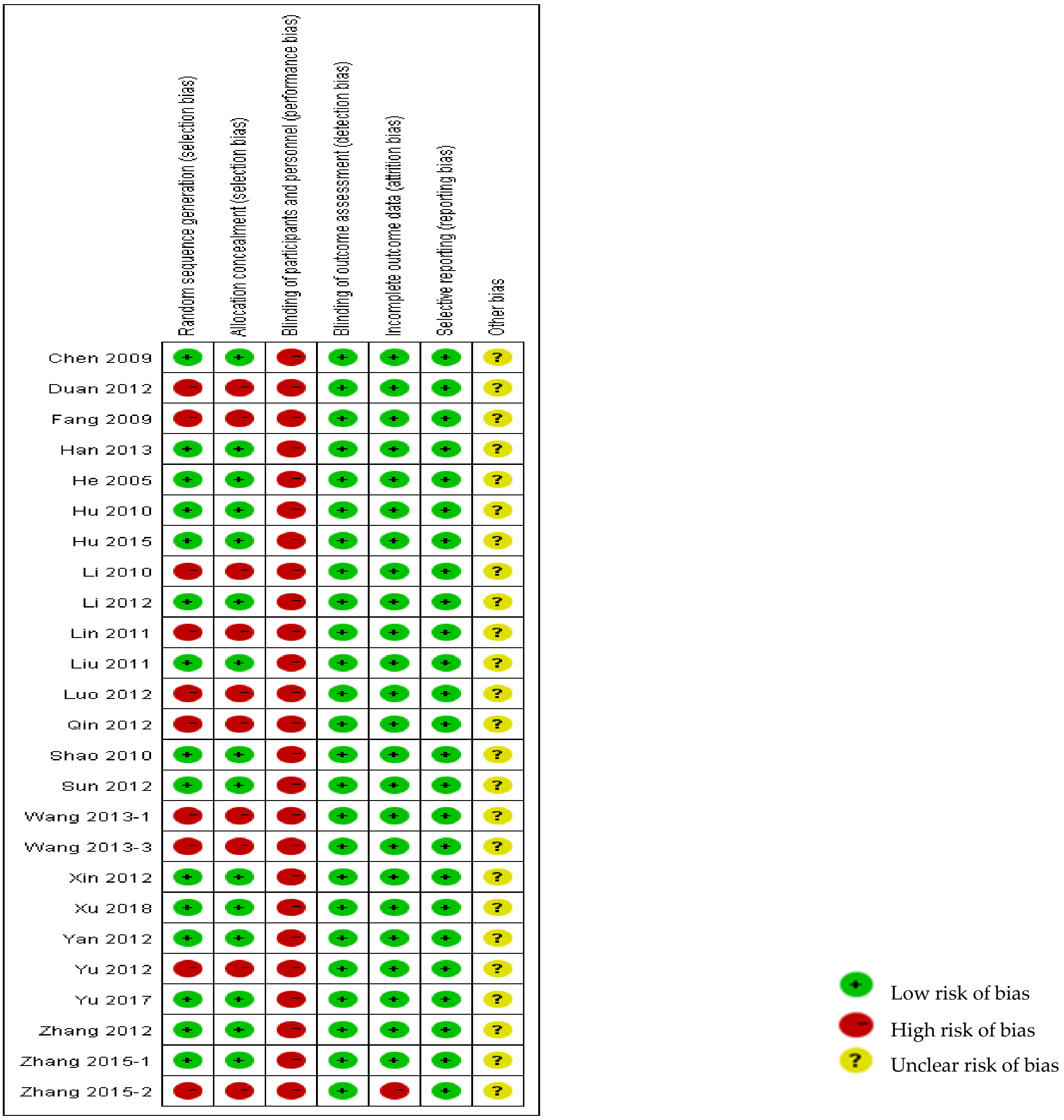

Figure S1. Summary chart of risk of bias assessment for included studies using the Cochrane risk of bias tool.

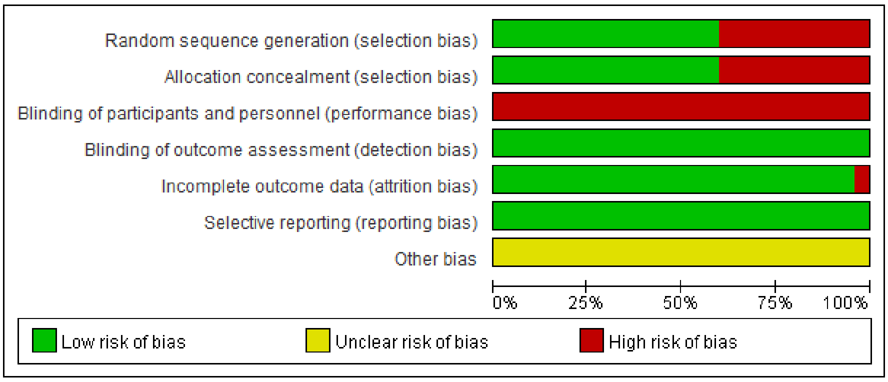

Figure S2. Risk of bias presented as percentages across all included studies.

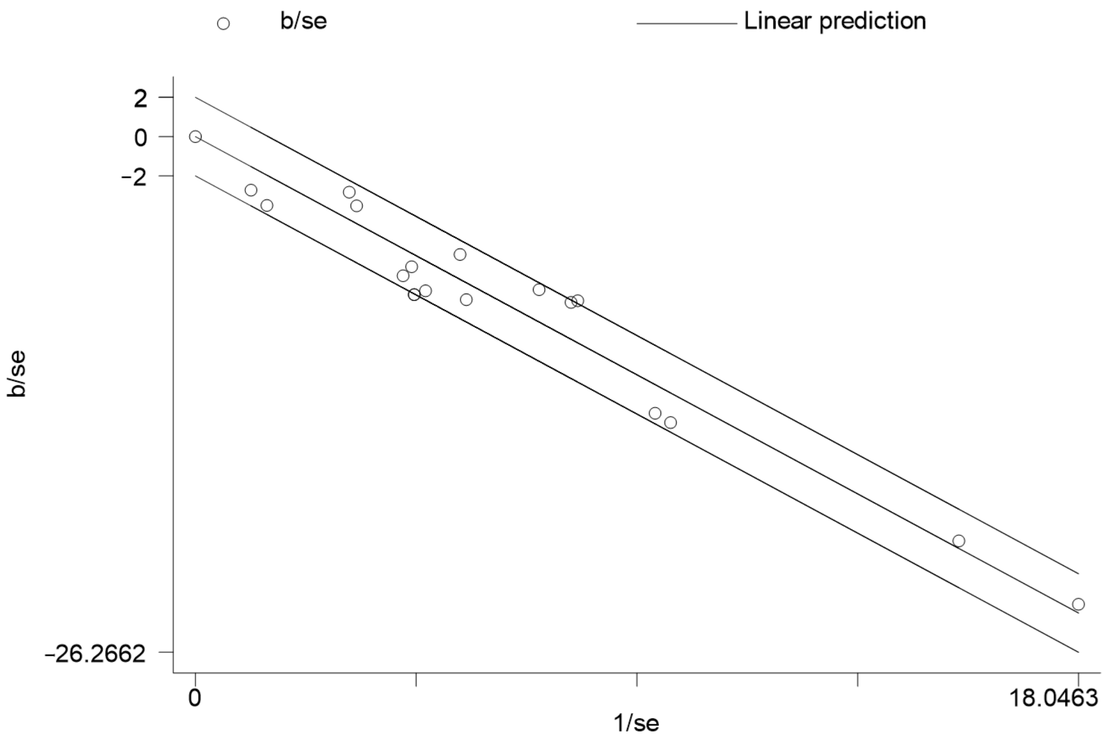

Figure S3. Galbraith plot of effects on LOS.

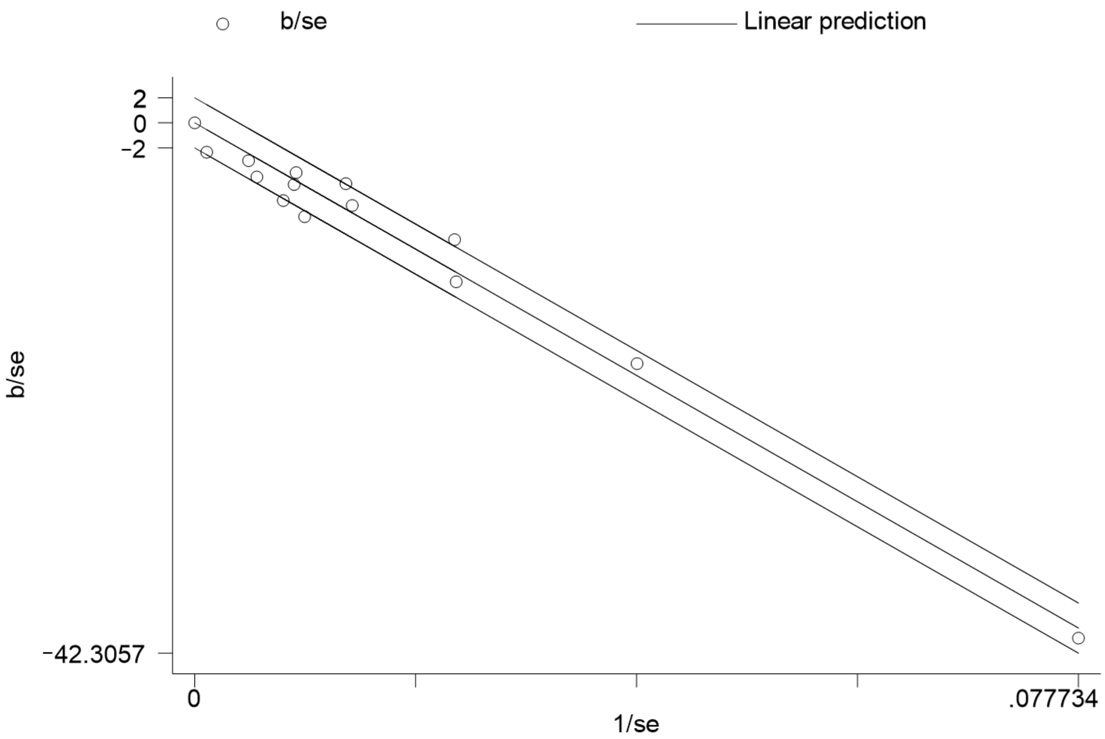

Figure S4. Galbraith plot of effects on DHC.

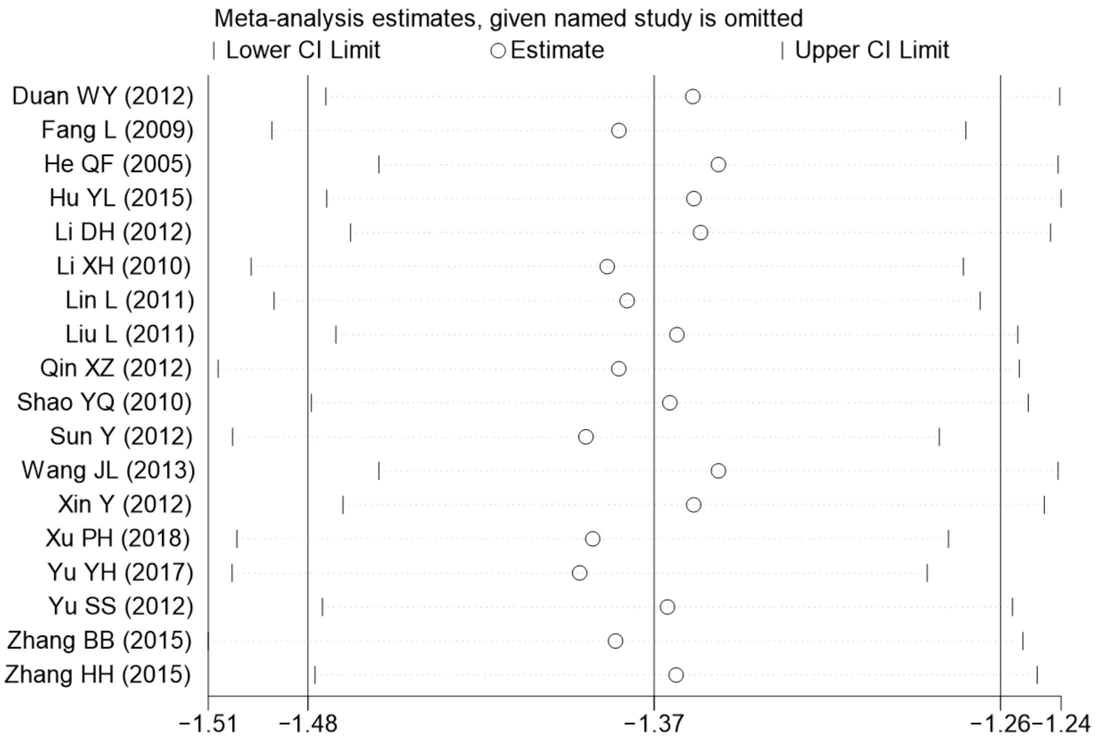

Figure S5. Sensitivity analyses for assessing the impact of individual studies on the pooled estimate of LOS.

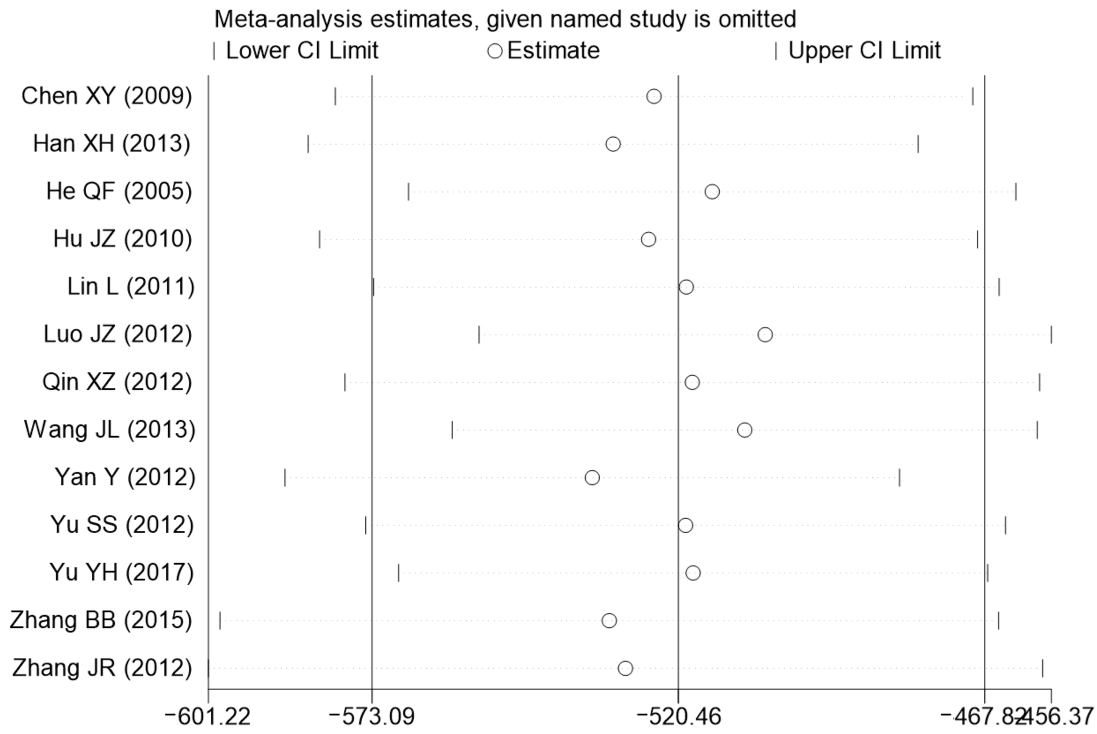

Figure S6. Sensitivity analyses for assessing the impact of individual studies on the pooled estimate of DHC.

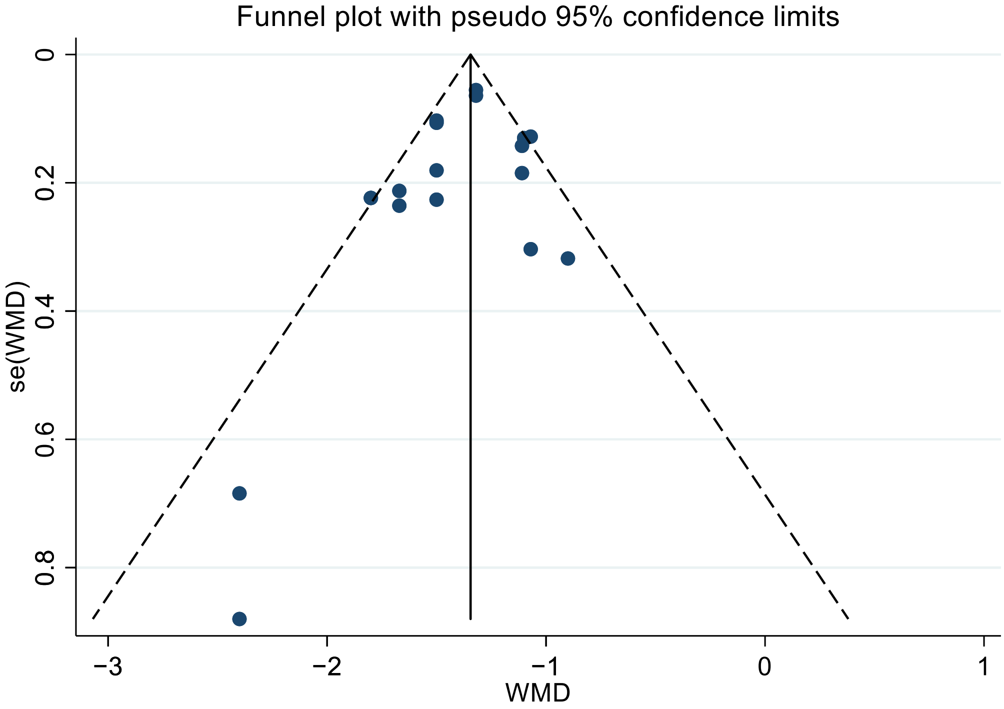

Figure S7. Metafunnel funnel plot for the effect of LOS.

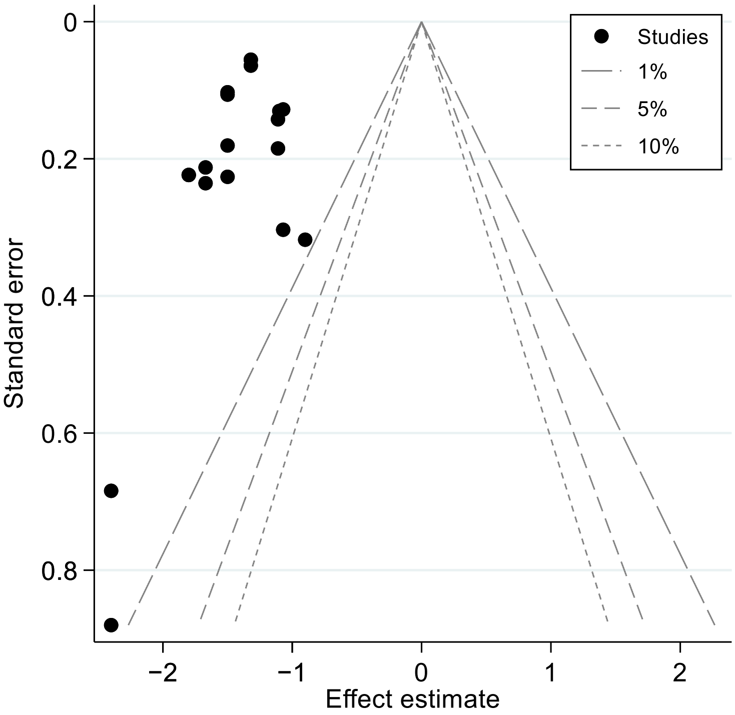

Figure S8. Confunnel funnel plot of the effect on LOS.

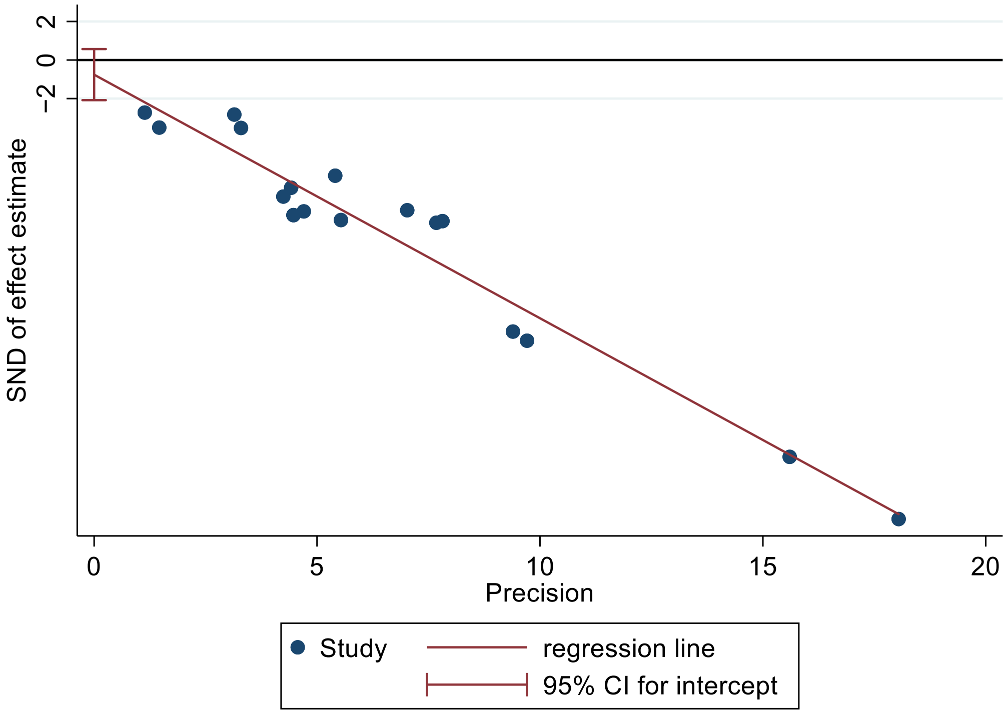

Figure S9. Egger's linear regression of effect on LOS.

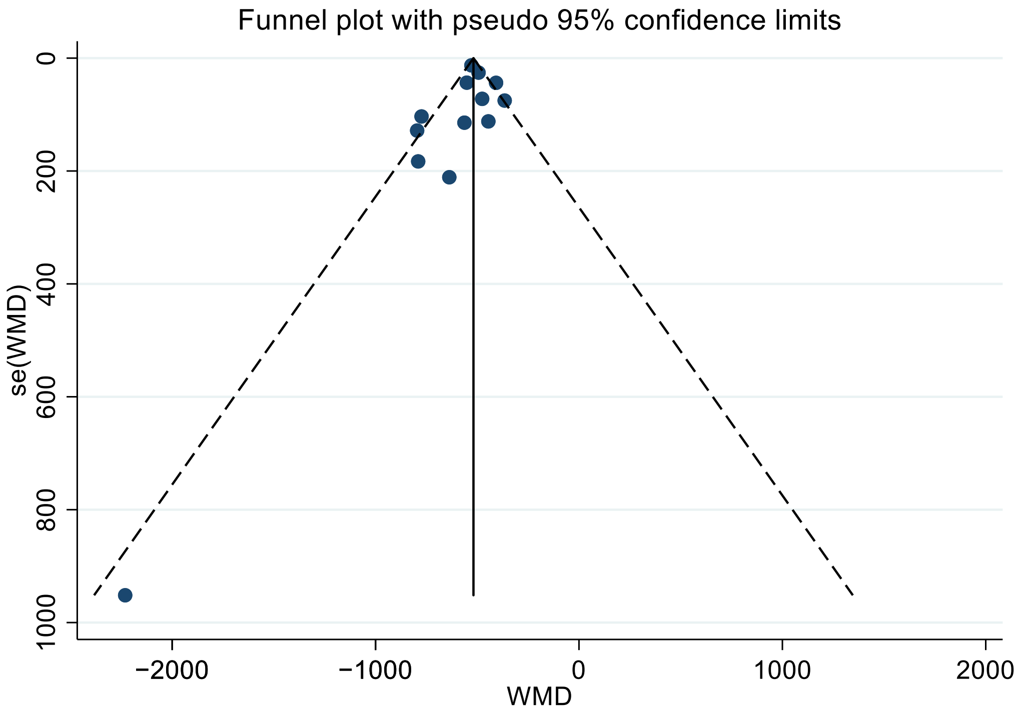

Figure S10. Metafunnel funnel plot for effect of DHC.

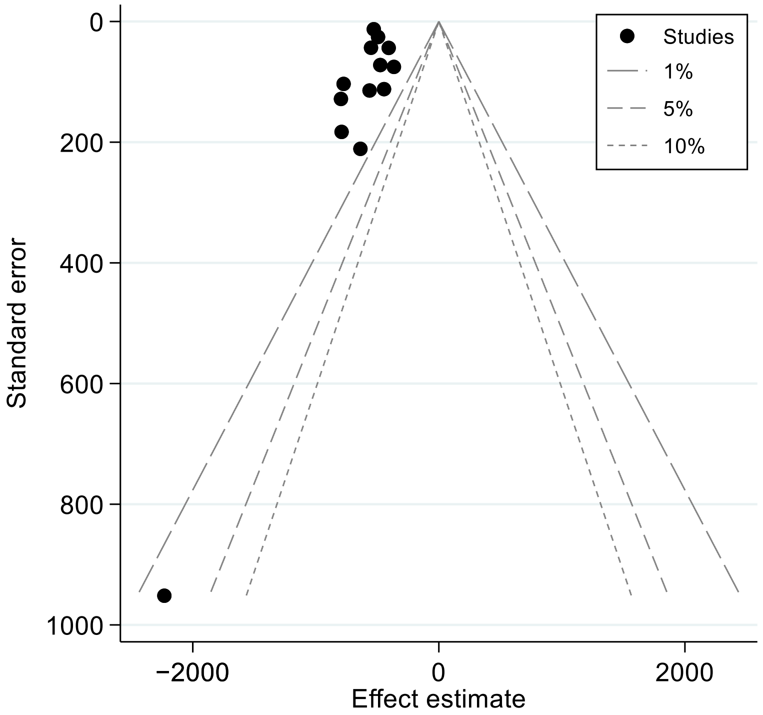

Figure S11. Confunnel funnel plot of effect on DHC.

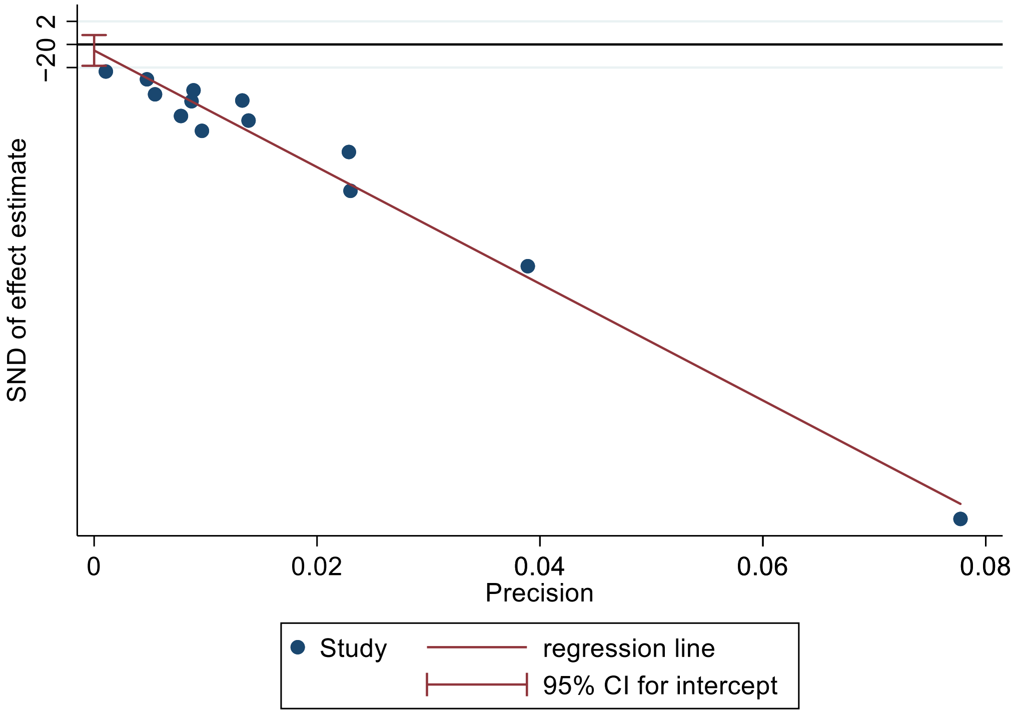

Figure S12. Egger's linear regression of effect on DHC.
